# Potential Role for HIV-Specific CD38^−^/HLA-DR^+^ CD8^+^ T Cells in Viral Suppression and Cytotoxicity in HIV Controllers

**DOI:** 10.1371/journal.pone.0101920

**Published:** 2014-07-07

**Authors:** Stéphane Hua, Camille Lécuroux, Asier Sáez-Cirión, Gianfranco Pancino, Isabelle Girault, Pierre Versmisse, Faroudy Boufassa, Olivier Taulera, Martine Sinet, Olivier Lambotte, Alain Venet

**Affiliations:** 1 INSERM U1012, Le Kremlin-Bicêtre, France; 2 Université Paris-Sud 11, Le Kremlin-Bicêtre, France; 3 Institut Pasteur, Unité de Régulation des Infections Rétrovirales, Paris, France; 4 INSERM U1018, Le Kremlin-Bicêtre, France; 5 Assistance Publique-Hôpitaux de Paris (AP-HP), Hôpital Bicêtre, Département d'épidémiologie, Le Kremlin-Bicêtre, France; 6 AP-HP, Hôpital Saint-Louis, Paris, France; 7 AP-HP, Hôpital Bicêtre, Service de Médecine Interne, Le Kremlin-Bicêtre, France; New York University, United States of America

## Abstract

**Background:**

HIV controllers (HIC) are rare HIV-1-infected patients who exhibit spontaneous viral control. HIC have high frequency of CD38^−^/HLA-DR^+^ HIV-specific CD8^+^ T cells. Here we examined the role of this subset in HIC status.

**Materials and Methods:**

We compared CD38^−^/HLA-DR^+^ CD8^+^ T cells with the classical CD38^+^/HLA-DR^+^ activated phenotype in terms of 1) their activation status, reflected by CD69, CD25, CD71, CD40 and Ki67 expression, 2) functional parameters: Bcl-2 expression, proliferative capacity, and IFN-γ and IL-2 production, and 3) cytotoxic activity. We also investigated how this particular profile is generated.

**Results:**

Compared to CD38^+^/HLA-DR^+^ cells, CD38^−^/HLA-DR^+^ cells exhibited lower expression of several activation markers, better survival capacity (Bcl-2 MFI, 367 [134–462] vs 638 [307–747], *P* = 0.001), higher frequency of polyfunctional cells (15% [7%–33%] vs 21% [16%–43%], *P* = 0.0003), greater proliferative capacity (0-fold [0–2] vs 3-fold [2]–[11], *P* = 0.007), and higher cytotoxicity *in vitro* (7% [3%–11%] vs 13% [6%–22%], *P* = 0.02). The CD38^−^/HLA-DR^+^ profile was preferentially generated in response to low viral antigen concentrations.

**Conclusions:**

These data highlight the role of CD38^−^/HLA-DR^+^ HIV-specific CD8^+^ T cell cytotoxicity in HIC status and provide insights into the mechanism by which they are generated. Induction of this protective CD8^+^ subset may be important for vaccine strategies.

## Introduction

A very small number of HIV-1-infected individuals spontaneously control viral replication. These so-called HIV controllers (HIC) usually have relatively high CD4^+^ T cell counts and remain AIDS-free for several decades [1], [2]. Several factors have been implicated in HIC status, including viral defects, cellular factors, and the innate immune system [3], but T cells, especially HIV-specific CD8^+^ T cells, are considered to play a major role. Overrepresentation of certain HLA class I alleles (especially HLA-B*57 and HLA-B*27) is associated with strong HIV-specific CD8^+^ T cell responses [4]–[6]. Studies of simian disease models also show that expression of the MHC class I alleles Mamu-B*08 and Mamu-B*17 correlates with viral control [7], [8]. Several studies have linked viral control to HIV-specific CD8^+^ T cell polyfunctionality [9], proliferation to HIV antigens, and lytic granule content [10], [11].

We and others have shown that HIC usually possess HIV-specific CD8^+^ T cells capable of suppressing HIV replication *ex vivo* [12], [13]. Although HIV-specific CD8^+^ T cells usually express similar levels of two activation markers – CD38 and HLA-DR – which correlate with viral load in non-controllers [14], they exhibit a peculiar activation phenotype in HIC, with low CD38 expression and high HLA-DR expression [13], [15]. Immune activation can have opposite effects on CD8^+^ T cell functional capacities. Indeed, a degree of immune activation is necessary for efficient effector responses (resting CD8^+^ T cells usually lack effector functions but can become cytotoxic when activated [16]), whereas overactivation may lead to exhaustion [17] and functional inefficiency [18]. The CD38^−^/HLA-DR^+^ phenotype might reflect a low level of activation and could thereby contribute to HIC status [13], [15], [19].

Here we studied CD38^−^/HLA-DR^+^ HIV-specific CD8^+^ T cells in a large population of HIC, focusing on functional activation status, memory/effector functions, and the mechanism underlying HLA-DR expression in the absence of CD38 expression.

## Materials and Methods

### Ethics statement

All the subjects provided their written informed consent to participation. The study was approved by the local investigational review board (*Comité de Protection des Personnes Ile de France VII*, Paris, France) and performed in accordance with the tenets of the Declaration of Helsinki.

### Study participants

We collected samples from 120 HIV-infected individuals, comprising 80 patients enrolled in the French ANRS CO21 CODEX cohort of HIC (inclusion criteria: HIV infection at least 5 years previously; 5 latest plasma HIV RNA values <400 copies/mL; no HAART), 21 untreated, chronically viremic patients, and 19 HAART-treated aviremic individuals (HIV RNA <50 copies/mL) enrolled in the French ANRS CO6 PRIMO cohort. Clinical and biological characteristics of the study participants are shown in Table 1. Peripheral blood samples from seven HIV-uninfected healthy blood donors (HD) were obtained from *Etablissement Français du Sang* (Paris, France).

**Table 1:** Study population.

	Viremic	HAART	HIC	*P*
n	21	19	80	
Age (years)	38 [31–46]	41 [34–44]	48 [42–53]	<0.0001
Gender (% men)	95%	84%	54%	0,0004
Time since HIV diagnosis (years)	1,5 [1–2]	4 [1–6]	13 [9–17]	<0.0001
CD4/µl	499 [374–697]	645 [500–818]	801 [645–1020]	<0.0001
CD8/µl	1150 [862–1609]	648 [492–747]	792 [610–951]	0,0001
Plasma HIV-RNA (log10 copies/ml)^a^	4,62 [4,44–4,81]	<1,7	1 [0,3–1,7]	<0.0001

Median values [1^st^–3^rd^ interquartile range] are shown for age, time since diagnosis, CD4^+^ and CD8^+^ T cell counts, and HIV RNA viral loads.

a Viremic and HAART-treated patients' RNA viral loads were measured using an assay with a quantification limit of <50 copies/ml, while values in HIC were obtained with ultrasensitive assays.

### Cell preparation

Peripheral blood mononuclear cells (PBMC) were isolated from EDTA-anticoagulated blood by Ficoll density gradient centrifugation and stored in liquid nitrogen. Human leukocyte antigen typing used the complement-dependent microlymphocytotoxic technique (Ingen).

### Flow cytometry of HIV-specific CD8^+^ T cells

Specific CD8^+^ T cells were characterized by staining, first for 15 minutes at room temperature with APC-labeled peptide-HLA class I multimers (Proimmune and Immudex for HLA-B*57 peptides) derived from the HIV proteins Gag, Nef, Pol, and Env and from the Epstein-Barr virus (EBV) proteins BMLF-1 and BZLF-1, and then with labeled antibodies for 15 minutes at 4°C. Anti-CD38-PerCP-Cy5.5 and anti-HLA-DR-PE-Cy7 were used to identify and sort the different activation subsets. The following antibodies were used to characterized HIV-specific CD8^+^ T cells: FITC–coupled anti-CD71, -CD40, -Bcl-2 and -Granzyme B; PE-coupled anti-Ki67, -CD69 and -CD25; V450-coupled anti-CD8 (BD Biosciences); VioGreen-coupled anti-CD3 (Miltenyi Biotec); and PE-coupled anti–perforin (clone D48, Diaclone). Dead cells were excluded by using the Live Dead Fixable Near-IR Dead Cell Stain kit (Molecular Probes, Invitrogen). For intracellular staining, cells were incubated with the appropriate antibodies for 30 minutes at 4°C after incubation with FACS permeabilizing solution (BD Biosciences). Samples were acquired on an LSR Fortessa cell analyzer (BD Biosciences) and analyzed with FACS DIVA software (BD Biosciences). An example of the gating strategy is shown in the Figure S1 and an example of the expression of the activation markers and cytokine production by the different subsets is shown in Figure S2. We assessed an average of two tetramers per patient and acquired at least 80 events per subset studied.

CD8^+^ T cells were sorted with an ARIA cell sorter (BD Biosciences) into the following activation subsets: CD38^−^/HLA-DR^+^, CD38^+^/HLA-DR^−^, and CD38^+^/HLA-DR^+^, using anti-CD56-PE to exclude NK cells, anti-CD8-V450 and anti-CD4-APC-H7 antibodies (BD Biosciences) according to the manufacturer's guidelines. After sorting, cells were resuspended at a density of 1×10^6^/mL and cultured for five days before used in the cytotoxicity assay described below. Purity was routinely >95% for each subset (data not shown). Too few cells expressed the CD38^+^/HLA-DR^−^ phenotype to obtain interpretable data.

### Intracellular cytokine production

PBMC were stimulated for 15 hours in medium containing the relevant optimal HIV peptide (2 µM). After 1 hour of stimulation, cytokine secretion was blocked by adding brefeldin A (10 µg/mL, Sigma-Aldrich Chemie). After further incubation, samples were stained as described above. Anti-IFN-γ-APC and anti-IL-2-PE antibodies (BD Biosciences) were used to detect intracellular cytokine production. A negative control (medium) and a positive control (staphylococcal enterotoxin B, SEB) were included in each experiment. The polyfunctionality was assessed by measuring the dual production of IFN-γ and IL-2. The results were expressed as frequency of IFN-γ^+^/IL-2^+^ producing cells among IFN-γ^+^ or IL-2^+^ producing cells.

### *In vitro* HIV suppression assay

The capacity of CD8^+^ T cells to suppress HIV-1 infection of autologous CD4^+^ T cells *ex vivo* was assessed as described in details elsewhere [20]. Briefly, CD4^+^ T cells were activated with phytohemagglutinin (PHA) and IL-2 for three days, then infected with HIV-1 BaL and cultured alone or with unstimulated autologous CD8^+^ T cells (1∶1 ratio). P24 antigen was measured in culture supernatants with an ELISA method (Zeptometrix) as a measure of viral replication. The capacity of CD8^+^ T cells to suppress HIV infection was expressed as the log decrease in p24 production when superinfected CD4^+^ T cells were cultured in the presence of CD8^+^ T cells.

### Cytotoxicity assay

The Grantoxilux Plus! cytotoxicity assay was used according to the manufacturer's instructions (OncoImmunin, Inc). It is based on Granzyme-B-mediated intracellular cleavage of a fluorogenic substrate in live target cells. Briefly, PBMC were used as target cells, CD8^+^ T cells as effectors, and CD8-depleted PBMC as feeders. Target cells were stimulated with PHA and IL-2 for five days. Effector cells were cocultured with feeders at a ratio of 1∶1 and with HIV peptide (2 µM) for five days. On day 5, target cells were primed for 1 h with the cognate or an irrelevant peptide, labeled with TFL4 (OncoImmunin, Inc.), and then cocultured with CD8^+^ T cells at an Effector:Target ratio of 50∶1. The cells were then incubated with Granzyme B substrate (OncoImmunin Inc.) for 1 h and analyzed immediately by flow cytometry. The results are expressed as the difference between the frequency of lysed target cells incubated with the cognate peptide and the frequency of lysed target cells incubated with the irrelevant peptide. An example of the cytotoxic assay is shown in Figure S3.

### Healthy donor cell culture

CD8^+^ T cells were isolated by using a human anti-CD56 antibody (Miltenyi Biotec) to deplete NK cells and a human anti-CD8 antibody (Miltenyi Biotec). Purity was greater than 95%. Isolated CD8^+^ T cells were cultured *in vitro* (2×10^6^ cells/mL) with medium alone, with the optimal concentrations of peptides, or with anti-CD3/CD28 antibodies as control (Miltenyi Biotec). IFN-α (PBL InterferonSource) was used at 500 IU/mL.

### Functional avidity

Functional avidity was measured in an IFN-γ ELISpot assay using single-epitope peptides corresponding to optimal HIV-CTL epitopes (National Institutes of Health HIV Molecular Immunology Database: http://www.hiv.lanl.gov/content/immunology/tables/optimal_ctl_ summary.html) according to the subjects' HLA types. Functional avidity of CD8^+^ T cell responses was assessed by performing limiting peptide dilutions ranging from 10^−5^ to 10^−11^ M in *in vitro* assays as described elsewhere [21]. It was defined as the peptide concentration required to achieve 50% of the maximal response (EC_50%_) and was expressed as log EC_50%_.

### Statistical methods

Data were analyzed with Prism software (GraphPad Software Inc.). Groups were compared using a nonparametric Mann-Whitney or Wilcoxon paired test for continuous data or a chi-squared test for categorical data. Correlations were evaluated using Spearman's rank correlation coefficient. The Spearman r correlation and (in the Figures) the Pearson correlation curve are indicated for significant correlations. The threshold for statistical significance was set to *P*<0.05.

## Results

### Study population

Baseline data for the 80 HIC, 21 viremic patients and 19 HAART-treated patients are shown in Table 1. HIC differed significantly from the other two groups of patients in terms of the sex ratio, age, CD4^+^ and CD8^+^ T cell counts, viral RNA loads, and time since diagnosis.

### Higher frequencies of CD38^−^/HLA-DR^+^ HIV-specific CD8^+^ T cells in HIV controllers

**Figure 1:**
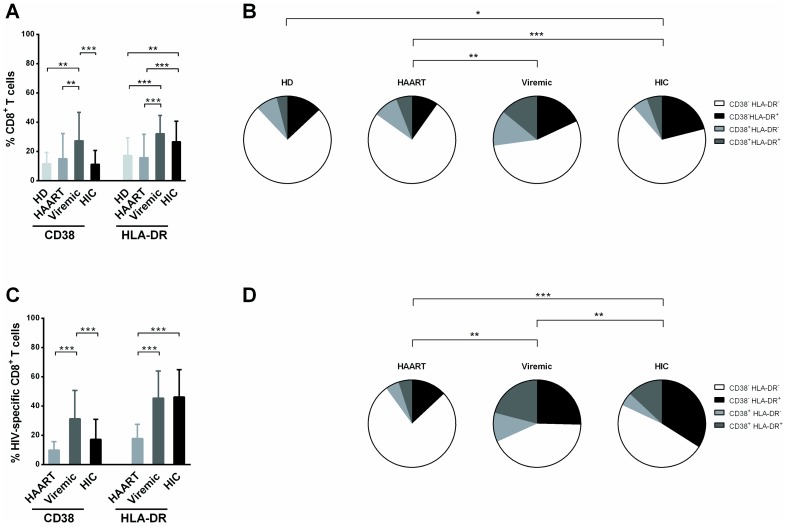
CD38 and HLA-DR expression on bulk and HIV-specific CD8^+^ T cells. (A) Proportions of bulk CD8^+^ T cells from HD (n = 16, light gray bars), HAART-treated patients (n = 19, mid gray bars), viremic patients (n = 21, dark gray bars) and HIC (n = 79, black bars) expressing CD38 and HLA-DR. (C) Proportions of HIV-specific CD8^+^ T cells from HAART-treated patients (n = 13, mid gray bars), viremic patients (n = 39, dark gray bars) and HIC (n = 80, black bars) expressing CD38 and HLA-DR. Pie charts representing CD38^−^/HLA-DR^−^ (white), CD38^−^/HLA-DR^+^ (black), CD38^+^/HLA-DR^−^ (light gray) and CD38^+^/HLA-DR^+^ (dark gray) cells among bulk (B) and HIV-specific CD8^+^ T cells (D). Statistical differences shown in the pie charts are based on the difference in the frequency of the CD38^−^/HLA-DR^+^ subset between the different groups. * *P*<0.05, ** *P*<0.01, *** *P*<0.001.

We first compared CD38 and HLA-DR expression *ex vivo* on bulk CD8^+^ T cells in the healthy donors (HD), viremic patients, HAART-treated patients, and HIV controllers (HIC) (Figure 1A–B) and on HIV-specific CD8^+^ T cells in the three groups of HIV-infected patients (Figure 1C–D). As previously reported by our group [15], median [1^st^–3^rd^ interquartile range] CD38 expression was lower in HD, HAART-treated patients and HIC (11% [6%–14%], 12% [5%–14%], 9% [5%–14%] respectively) than in viremic patients (20% [14%–39%]) (*P* = 0.0001 for each comparison, Fig. 1A). HLA-DR expression was higher in viremic patients and HIC (29% [23%–38%] and 27% [19%–32%], respectively) than in HD and HAART-treated patients (14% [11%–21%] and 11% [7%–19%]) (*P* = 0.0002 and *P*<0.0001 for viremic patients versus HD and HAART, respectively; *P* = 0.006 and *P* = 0.0008 for HIC versus HD and HAART, respectively; Fig. 1A). Consequently, the CD38^+^/HLA-DR^+^ phenotype was more frequent in viremic patients (14% [8%–19%]) than in HD, HAART-treated patients and HIC (2% [1%–3%], 3% [1%–4%] and 4% [3%–8%], respectively) (*P*<0.0001 for all three comparisons, Fig. 1B). CD38^−^/HLA-DR^+^ expression was more frequent in HIC than in HAART-treated patients (21% [15%–28%] vs 9% [5%–12%], *P* = 0.0003, Fig. 1B).

HIV-specific CD8^+^ T cell frequency was higher in HIC (2705 SFC/10^6^ PBMC [1135–5675]) and in viremic patients (2255 SFC/10^6^ PBMC [1334–6933]) than in HAART-treated patients (1138 SFC/10^6^ PBMC [179–2733]) (*P = *0.009 and *P = *0.03 between HIC and HAART-treated patients and between viremic patients and HAART-treated patients respectively). HIV-specific CD8^+^ T cells with high CD38 expression were also less frequent in HAART-treated patients and HIC (10% [6%–16%] and 13% [11%–39%], respectively) than in viremic patients (28% [15%–48%]) (*P*<0.0001 for both comparisons, Fig. 1C).

HLA-DR expression in HAART-treated patients (19% [10%–24%]) was far lower than in HIC (47% [32%–61%], *P*<0.0001), and in viremic patients (50% [32%–58%], Fig. 1C). The frequency of CD38^−^/HLA-DR^+^ was thus higher in HIC (33% [20%–46%]) than in both HAART-treated and viremic patients (13% [6%–19%] and 20% [15%–47%], respectively; *P*<0.0001 for both comparisons, Figure 1D). Moreover, there is a correlation between the frequency of CD38^−^/HLA-DR^+^ and the frequency of HIV-specific CD8^+^ T cells (*P* = 0.002, r = 0.22) which suggested that CD38^−^/HLA-DR^+^ may contribute mainly to the high frequency of HIV-specific CD8^+^ T cells

### CD38^−^/HLA-DR^+^ HIV-specific CD8^+^ T cells are more weakly activated than their CD38^+^/HLA-DR^+^ counterparts

**Figure 2:**
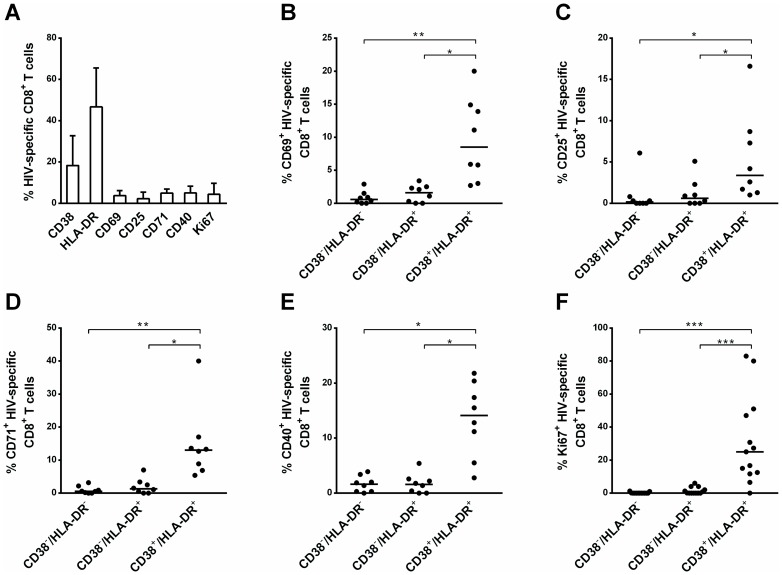
Activation phenotype of CD38^−^/HLA-DR^−^, CD38^−^/HLA-DR^+^ and CD38^+^/HLA-DR^+^ subsets from HIC. (A) Proportions of HIV-specific CD8^+^ T cells expressing CD38, HLA-DR, CD69, CD25, CD71, CD40 and Ki67. (B–F) Proportions of CD38^−^/HLA-DR^−^, CD38^−^/HLA-DR^+^ and CD38^+^/HLA-DR^+^ HIV-specific CD8^+^ T cells expressing CD69 (B), CD25 (C), CD71 (D), CD40 (E) and Ki67 (F) (n = 8). * *P*<0.05, ** *P*<0.01, *** *P*<0.001.

Although expression *ex vivo* of the activation markers CD69 (activation marker), CD40 (costimulatory receptor), CD71 (transferrin receptor protein 1), CD25 (IL-2Rα) and Ki67 (proliferative marker) was rather low on HIV-specific CD8^+^ T cells in HIC (3% [2%–6%], 4% [2%–8%], 4% [3%–7%], 1% [1%–2%] and 1% [0%–9%], respectively, Fig. 2A), classical CD38^+^/HLA-DR^+^ activated cells exhibited higher expression of CD69 (9% [4%–15%]), CD40 (14% [7%–20%]), CD71 (13% [7%–16%]), CD25 (3% [1%–8%]) and Ki67 (25% [12%–49%]), than the other subsets. Strikingly, expression of these markers on the CD38^−^/HLA-DR^+^ subset was very low and similar to that observed on resting CD38^−^/HLA-DR^−^ cells (median 0–2% for both subsets and all activation markers). Thus, the CD38^−^/HLA-DR^+^ subset is more weakly activated than the CD38^+^/HLA-DR^+^ subset.

### The CD38^−^/HLA-DR^+^ subset contains a higher frequency of polyfunctional cells and has higher proliferative and cytotoxic capacities than the CD38^+^/HLA-DR^+^ subset

**Figure 3:**
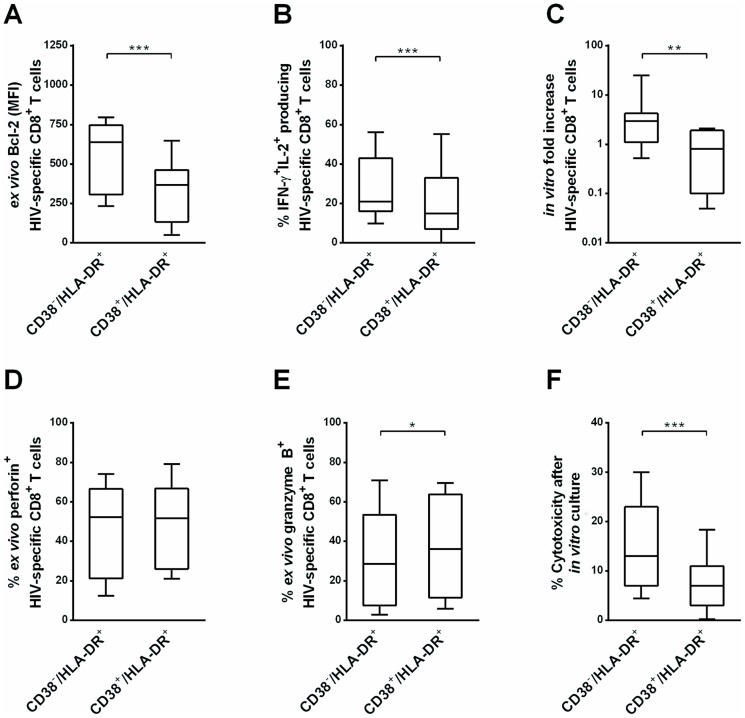
Qualitative features of CD38^−^/HLA-DR^+^ and CD38^+^/HLA-DR^+^ HIV-specific CD8^+^ T cell subsets in HIC. (A) Bcl-2 expression on CD38^−^/HLA-DR^+^ and CD38^+^/HLA-DR^+^ HIV-specific CD8^+^ T cells (n = 11). (B) Proportion of CD38^−^/HLA-DR^+^ and CD38^+^/HLA-DR^+^ HIV-specific CD8^+^ T cells producing both IFN-γ and IL-2 among HIV-specific CD8^+^ T cells producing IFN-γ or IL-2 (n = 35). (C) Fold increase in CD38^−^/HLA-DR^+^ and CD38^+^/HLA-DR^+^ HIV-specific CD8^+^ T cell numbers after 5 days of culture with HIV peptides (2 µM) (n = 7). (D–E) Proportion of CD38^−^/HLA-DR^+^ and CD38^+^/HLA-DR^+^ HIV-specific CD8^+^ T cells producing perforin (D) and granzyme B (E) (n = 35). (F) Graphs representing percentage cytotoxicity (measured as granzyme-B-mediated intracellular cleavage of a fluorogenic substrate) of CD38^−^/HLA-DR^+^ and CD38^+^/HLA-DR^+^ HIV-specific CD8^+^ T cells (n = 11). * *P*<0.05, ** *P*<0.01, *** *P*<0.001.

The survival capacity was assessed by measuring the difference of the median fluorescence intensity of Bcl-2 expression of one subset and the isotype. Bcl-2 expression *ex vivo*, was higher for CD38^−^/HLA-DR^+^ cells than for CD38^+^/HLA-DR^+^ cells (638 [307–747] vs 367 [134–462], *P* = 0.001, respectively, Fig. 3A).

We then analyzed dual production *in vitro* of IFN-γ and IL-2 as a marker of polyfunctionality. As shown in Figure 3B the CD38^−^/HLA-DR^+^ subset contained more IFN-γ^+^IL-2^+^-producing cells than the CD38^+^/HLA-DR^+^ subset (21% [16%–43%] vs 15% [7%–33%], *P* = 0.0003, Fig. 3B). We analyzed the proliferation capacity *in vitro* by assessing the number of HIV-specific CD8^+^ T cells at the beginning and the end of the culture and calculated the fold increase of HIV-specific CD8^+^ T cells. After sorting and culture with cognate peptides, proliferation of CD38^−^/HLA-DR^+^ cells increased 3.2-fold [1.2–7.8], whereas CD38^+^/HLA-DR^+^ cells did not proliferate significantly (0.4-fold [0.0–1.7]) (*P* = 0.007, Fig. 3C).

Although *ex vivo* perforin expression did not differ between the two subsets (52% [21%–67%] and 52% [26%–67%], respectively, *P* = 0.26, Fig. 3D), CD38^−^/HLA-DR^+^ cells exhibited lower granzyme B *ex vivo* expression than CD38^+^/HLA-DR^+^ cells (29% [8%–54%] and 36% [12%–64%], respectively, *P* = 0.01, Fig. 3E). However, *ex vivo* after sorting cells, cytotoxicity was very low and similar with the two subsets (0% [0%–8%] vs 4% [4%–16%], respectively, *P* = 0.2; data not shown). After sorting and coculture with CD8-depleted PBMC for 5 days, CD38^−^/HLA-DR^+^ cells showed increased cytotoxicity (13% [7%–23%]), contrary to the CD38^+^/HLA-DR^+^ subset (7% [3%–11%]) (*P* = 0.001, Fig. 3F). CD38^−^/HLA-DR^+^ CD8^+^ T cells thus responded significantly more strongly to cognate antigen than did their CD38^+^/HLA-DR^+^ counterparts.

*Ex vivo* HIV-suppressive activity was routinely evaluated with freshly isolated whole-blood T cells which often include several HIV specificities while cytotoxicity was evaluated only on immunodominant peptides. Of note, HIV-specific CD8^+^ T cell cytotoxicity correlated with HIV-suppressive activity, expressed as the log decline in p24 antigen (*P* = 0.004; r = 0.37, data not shown). Interestingly, only the frequency of the CD38^−^/HLA-DR^+^ HIV-specific CD8^+^ T cells correlated strongly with the log p24 decrease (*P*<0.0001; r = 0.32, data not shown). No such correlation was found with CD38^+^/HLA-DR^+^ cells (*P* = 0.5).

### The conditions of stimulation determine CD38 and HLA-DR expression by specific CD8^+^ T cells

**Figure 4:**
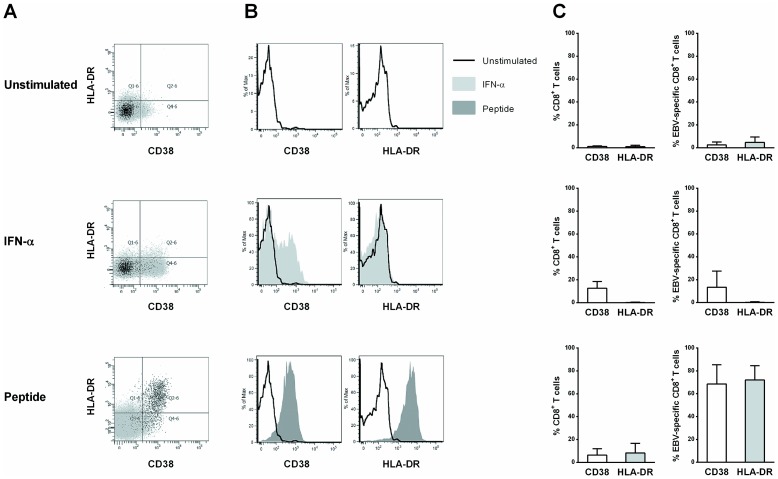
Influence of stimulatory conditions on the activation phenotype of specific CD8^+^ T cells. (A) Representative dot plots of HLA-DR and CD38 expression in unstimulated conditions (upper graph), after IFN-α stimulation (middle graph) or peptide stimulation (2 µM, lower graph) among specific (dark dots) and non-specific (gray dots) CD8^+^ T cells from healthy donors after a four-day culture period. CD38 and HLA-DR expression on bulk (C left panel) and specific CD8^+^ T cells (B and C right panel). (B) Flow cytometry histograms showing representative results for cells from one individual in unstimulated conditions (dark lines), after IFN-α stimulation (light gray histograms) or peptide stimulation (dark gray histograms). (C) Surface expression of CD38 (white bars) and HLA-DR (gray bars) on bulk and specific CD8^+^ T cells (n = 4).

Finally, we examined the mechanisms by which resting CD38^−^/HLA-DR^−^ cells become CD38^−^/HLA-DR^+^ cells in the context of viral infection. For this purpose, we used an *in vitro* resting and fully unactivated model in which EBV-specific CD8^+^ T cells which are mostly CD38^−^/HLA-DR^−^ as shown in Figure 4 are stimulated by EBV peptides in various conditions.

We postulated that the CD38^+^/HLA-DR^+^ profile might result from a dual activation pathway, in which antigenic peptide stimulation leads to HLA-DR expression while indirect activation by IFN-α leads to CD38 expression [22], [23]. As expected, IFN-α stimulation led to CD38 expression on bulk CD8^+^ T cells (14% [7%–17%]) and, to a lesser degree, on EBV-specific CD8^+^ T cells (9% [4%–28%]), whereas neither bulk nor EBV-specific CD8^+^ T cells expressed HLA-DR (0% [0%–1%] and 0% [0%–1%] respectively; Figure 4, middle panel), confirming the non-specific nature of IFN-α-induced activation. However, stimulation with EBV peptides led to strong expression of both CD38 and HLA-DR by EBV-specific cells (69% [53%–84%] vs 72% [61%–83%], respectively; Figure 4 lower panel), while no change was observed in the total CD8^+^ T cell population. We suspected that the increase in CD38 expression might be related to IFN-α production by non T cells in this model, but neither blockade with an anti-IFN-αR antibody nor the use of highly purified CD8^+^ T cells significantly modified our results.

**Figure 5:**
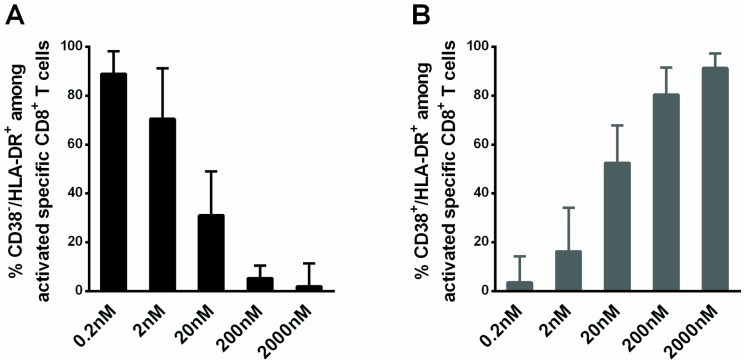
Stimulation with a low antigen concentration induces the CD38^−^/HLA-DR^+^ phenotype on specific CD8^+^ T cells. (A) Graphs representing the frequency of (A) CD38^−^/HLA-DR^+^ cells (dark histograms) and (B) CD38^+^/HLA-DR^+^ cells (dark gray histograms) among activated EBV-specific healthy donor CD8^+^ T cells (i.e. those expressing CD38 and/or HLA-DR) after a four-day culture period (n = 8).

Interestingly, the antigen concentration correlated positively with the degree of activation and also affected the profile of activated cells. At low antigen levels, most activated cells expressed HLA-DR but not CD38. Indeed, the frequency of CD38^−^/HLA-DR^+^ cells among activated cells was higher at low antigen levels (89% [44%–98%] and 2% [1%–11%] at 0.2 nM and 2000 nM, respectively; *P* = 0.0006, Fig. 5A), whereas CD38^+^/HLA-DR^+^ cells exhibited the opposite behavior (4% [0%–14%] vs 91% [78%–97%] at 0.2 nM and 2000 nM, respectively; *P* = 0.001, Fig. 5B).

### The CD38^−^/HLA-DR^+^ phenotype by specific CD8^+^ T cells in HIC is conditioned by low viral loads and high antigen sensitivity

**Figure 6:**
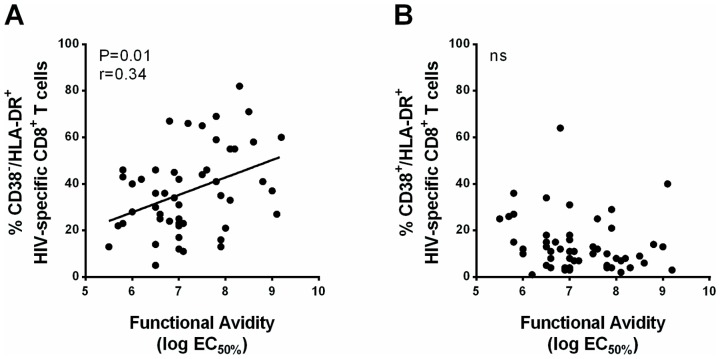
High antigen sensitivity is associated with a high frequency of CD38^−^/HLA-DR^+^ cells in HIC. The antigen sensitivity of HIV-specific CD8^+^ T cells was measured in ELISpot assays with serial limiting dilutions of antigenic peptides (from 10^−5^ to 10^−11^ M) and was expressed as the log molar concentration of peptide yielding 50% of the maximum response (EC_50%_). Correlations between the proportion of CD38^−^/HLA-DR^+^ (A) or CD38^+^/HLA-DR^+^ (B) and the antigen sensitivity of HIV-specific CD8^+^ T cells from HIC. Correlations were evaluated using the Spearman rank correlation coefficient. The Spearman r correlation and the Pearson correlation curve are indicated for significant correlations (n = 47).

HIV-specific CD8^+^ T cell sensitivity to cognate antigen, measured with an ELISpot assay, showed an overall positive correlation between the frequency of *ex vivo* CD38^−^/HLA-DR^+^ in these cells and the antigen sensitivity of HIV-specific CD8^+^ T cells (r = 0.34, *P* = 0.01, Figure 6A), whereas no such correlation was seen with CD38^−^/HLA-DR^−^ cells (*P* = 0.39) or CD38^+^/HLA-DR^+^ cells (*P* = 0.05, Figure 6B).

These results suggest strongly that the CD38^−^/HLA-DR^+^ subset is induced in the context of low viral loads and expressed on highly avid cells.

## Discussion

HIV-specific CD8^+^ T cells in HIV controllers were initially shown to exhibit (i) efficient proliferation and cytotoxicity [10], [11], (ii) strong virus suppression *ex vivo* [13], and (iii) a particular activation phenotype: CD38^−^/HLA-DR^+^ [13], [15]. Little is known about this CD8^+^ T cell subset and its possible role in HIC. The activation status of this CD38^−^/HLA-DR^+^ subset in HIC is puzzling, as HLA-DR expression suggests an activated state, while the lack of CD38 expression suggests the opposite. None of the other activation markers studied here were expressed by these cells, including those expressed early after activation, such as CD69 and CD25, and those expressed later, such as CD71 and CD40 [24], [25]. This exclusive HLA-DR expression suggests a low state of activation, and is in keeping with absent expression of Ki-67, a marker of cell cycling. A previous study showed coexpression of HLA-DR and Ki-67 by these cells after immunotherapy, but the authors did not evaluate CD38 expression [26].

This low level of activation is compatible with efficient survival evaluated here in terms of Bcl-2 expression level, and also with good proliferative potential. Previous studies showed an association between HLA-DR expression and proliferation [26], [27]. And indeed, these CD38^−^/HLA-DR^+^ cells expanded 3-fold in culture, whereas their CD38^+^/HLA-DR^+^ counterparts did not expand significantly. Of note, this expansion of CD38^−^/HLA-DR^+^ cells was associated with differentiation into effector cells, as their HIV-specific cytotoxicity was higher than that of CD38^+^/HLA-DR^+^ cells, even after adjusting for the HIV-specific-effector-to-target ratio. These data confirm those reported by Blankson et al., who showed that sorted CD38^−^/HLA-DR^+^ CD8^+^ T cells from HIC, when placed in short-term culture with autologous unstimulated CD4^+^ T cells, were the first cells to suppress viral replication [28]. Additionally, although CD38^−^/HLA-DR^+^ and CD38^+^/HLA-DR^+^ showed similar *ex vivo* cytotoxic capacity, the very low frequency of CD38^+^/HLA-DR^+^ cells, and their poor proliferative capacity shown here and elsewhere [29], make it unlikely that this population has a major role in suppressing viral replication. These data suggest that *in vivo* CD38^−^/HLA-DR^+^ cells which are less susceptible than CD38^+^/HLA-DR^+^ cells to exhaustion, have a greater capacity to control viral replication than CD38^+^/HLA-DR^+^ cells. Although we and others have observed that HIV-specific CD8^+^ T cells from some HIC patients lack HIV-suppressive activity *ex vivo*, they are capable of differentiating when exposed to cognate antigens, acquiring the ability to lyse infected cells and to suppress HIV replication [30].

We also explored the conditions in which the CD38^−^/HLA-DR^+^ phenotype is generated by using an *in vitro* model with EBV peptides and cells from healthy donors. We needed a non-activated condition to evaluate the mechanism of CD38 and HLA-DR expression. EBV infection is a model of infection with effective memory resting cells while HIV infection leads to persistent activation and inflammation even in HIC. Therefore, we believed that the EBV-model is a more appropriate model to address the question of CD38 and HLA-DR induction after activation. We confirmed that IFN-α induces CD38 expression but not HLA-DR expression [22], [23] and that this activation is not virus-specific. Conversely, EBV peptide stimulation led to activation of EBV-specific CD8^+^ T cells expressing both CD38 and HLA-DR. It has been shown that strong TCR stimulation by anti-CD3/CD28 leads to *cd38* transcription in purified CD8^+^ T cells [23], possibly explaining how CD38 is expressed following peptide stimulation even in IFN-αR-antagonizing conditions. However, we found that low EBV peptide concentrations elicited the CD38^−^/HLA-DR^+^ phenotype on the vast majority of activated cells, in keeping with the low viral load and lack of IFN-α production in experimental models of viral control [31]. Although plasmacytoid dendritic cells from HIC are able to produce IFN-α efficiently after HIV antigen stimulation *in vitro* [32], [33], HIC do not produce detectable IFN-α *in vivo*, as shown by tissue studies [34], which can explain the absence of IFN-α pathway mediated CD38 expression. The discrepancy between the low frequency of CD38^−^/HLA-DR^+^ cells among virus-specific CD8^+^ T cells in our *in vitro* model and the high frequency observed *in vivo* in HIC may be due to the contrast between brief activation *in vitro* and chronic activation by low HIV viral loads in HIC. The preferential activation of the CD38^−^/HLA-DR^+^ subset by low antigen concentrations is particularly interesting, as memory cells activated by low antigen concentrations show little senescence, have high proliferation rates, are able to persist for long periods [35], and maintain their TCR on the membrane surface, enabling a persistent cytotoxic response [36]. Furthermore, the peptide concentrations that yielded the CD38^−^/HLA-DR^+^ phenotype in our *in vitro* EBV model are similar to the estimated concentrations of HIV antigens present *in vivo* [37], [38]. In addition, the high antigen sensitivity observed in HIC may reinforce preferential induction of the CD38^−^/HLA-DR^+^ profile on the most sensitive cells, which are endowed with the best functional profile [21], [38]–[40]. Indeed, antigen sensitivity has been shown to correlate negatively with HIV cellular viral load [40]. However, high antigen sensitivity is not the only feature that can explain HIC status. Indeed, we observed no increase in the frequency of CD38^−^/HLA-DR^+^ cells in patients positive for HLA-B*27 or HLA-B*57 ([21] and data not shown) whereas antigen sensitivity is particularly high in this context [21], [39]. We moreover observed no difference in the frequency of this subset when comparing different HIV peptide specificities (data not shown). In addition, we have previously reported a lack of difference in perforin expression and inhibition of viral replication between HIC with and without protective HLA alleles [21], [41].

T cell activation is necessary for effector functions such as cytotoxicity and suppression of viral replication, but the persistent immune activation associated with systemic inflammation is known to play a key role in HIV disease progression [42], [43]. This is also the case in HIC, who display low but higher frequencies of classical activated CD38^+^/HLA-DR^+^ CD8^+^ T cells than do healthy donors (HD) and HAART-treated patients [43]. This excessive immune activation may result from the persistence of extremely low levels of HIV replication [44] and/or enhanced microbial translocation from the gut [45]. HIC exhibit a degree of disease progression and sometimes experience a decline in CD4^+^ T cells or even lose their ability to control HIV [46], possibly as a result of persistent immune activation [46], [47]. Alternatively, CD4^+^ T cell activation might permit persistent low-level infection of these cells, in turn maintaining efficient stimulation of the HIV-specific immune response [21], [43] and thereby helping to control viral replication through inducing CD38^−^/HLA-DR^+^ profile.

## Conclusions

The paradoxical activation profile of some HIV-specific CD8^+^ T cells, with HLA-DR but not CD38 expression and weak expression of all other activation markers, might confer the capacity to differentiate into effective cytotoxic cells after moderate activation [28] while avoiding the deleterious effects of excessive immune activation. It would be a favorable condition as compared to HAART-treated patients who display a very low viral load which leads to an absence of immune activation and effective response and as compared to viremic patients who display high viral load which leads to excessive activation and exhaustion. A high frequency of CD38^−^/HLA-DR^+^ cells among total CD8^+^ T cells has been linked to slow HIV disease progression and high CD4^+^ T cell counts [48]. We suspect that the expansion of this particular activated subset will lead to effective HIV-specific CD8^+^ T cells capable of rapid viral suppression. Induction of this protective cell subset by activation with low concentrations of HIV antigens, together with limited lymphocyte activation, might have implications for HIV vaccine strategies.

## Supporting Information

Figure S1 **Example of gating strategy of subsets of HIV-specific CD8^+^ T cells in HIV controllers.** Cells were gated (A) on live cells using live/dead stain cells kit, (B) on lymphocytes, (C) on CD3^+^/CD8^+^ expressing cells, (D) on HIV-specific CD8^+^ T cells using tetramer staining, (E) on different subset of CD38 and HLA-DR expressing cells using isotypes as control.
(TIF)

Figure S2 **Dot plots of activation marker expression and cytokine production on different subsets of HIV-specific CD8^+^ T cells in HIV controllers.** Representative dot plots of CD25 and CD40 (A), CD69 and CD71 (B), Ki67 (C) expression and IL-2 and/or IFN-γ secretion (D) on CD38^−^/HLA-DR^−^ (left panel), CD38^−^/HLA-DR^+^ (middle panel) and CD38^+^/HLA-DR^+^ (right panel) subsets
(TIF)

Figure S3 **Dot plots of the cytotoxic assay of CD38^−^/HLA-DR^+^ and CD38^+^/HLA-DR^+^ HIV-specific CD8^+^ T cells.** Cytotoxic capacity was assessed by measuring the frequency of positive target cells for granzyme B substrate. The results were expressed as the difference between the frequency using effector cells co-cultured with target cells incubated with the relevant peptide (B and D) and the frequency in the negative control using effector cells co-cultured with target cells incubated with irrelevant peptide (A and C).
(TIF)
