# Supplementary figures and images for: Potential Role for HIV-Specific CD38−/HLA-DR+ CD8+ T Cells in Viral Suppression and Cytotoxicity in HIV Controllers
Source: PLoS One. 2014 Jul 7;9(7):e101920. doi: 10.1371/journal.pone.0101920 (PMC4084978; doi:10.1371/journal.pone.0101920)

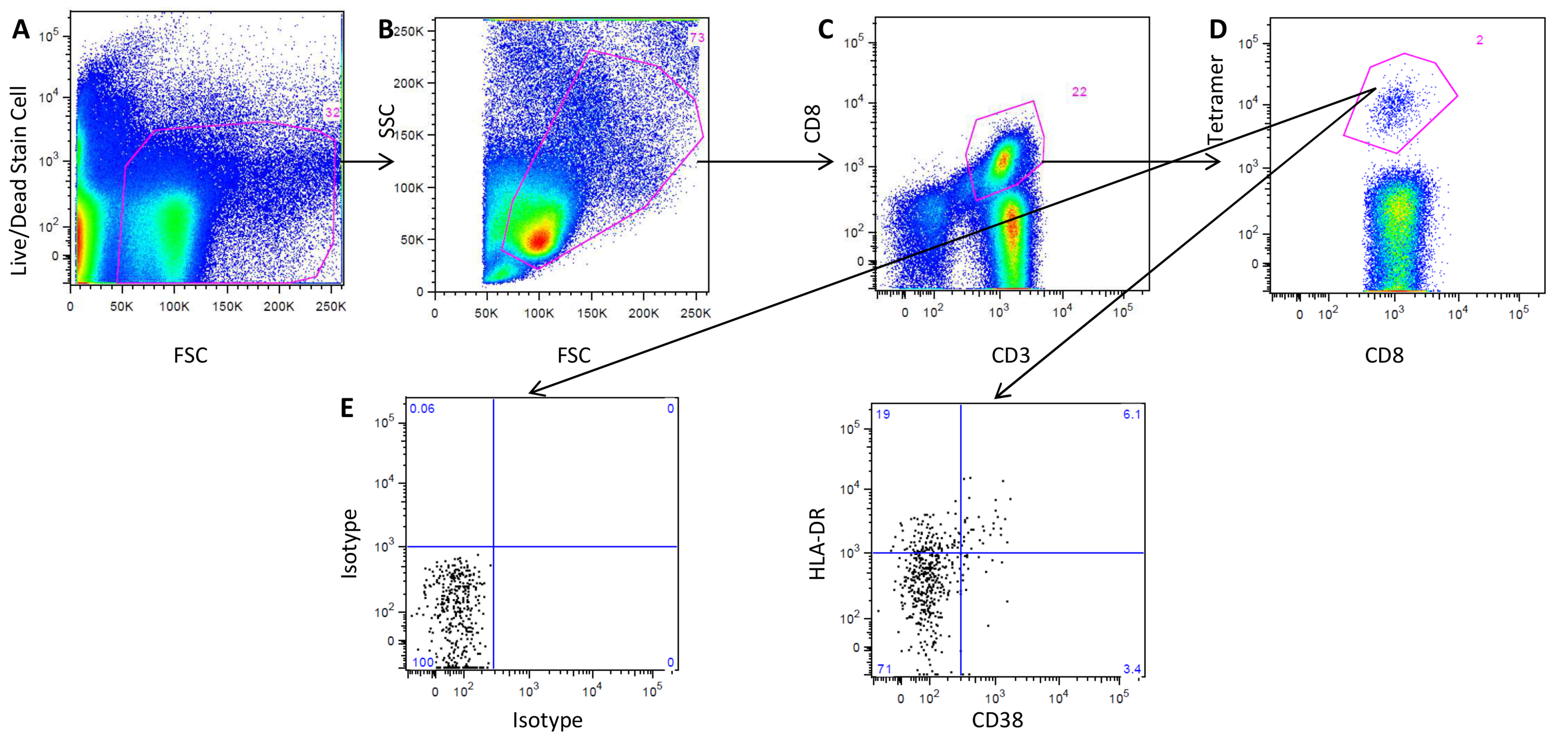

Supplement: Figure S1 — Example of gating strategy of subsets of HIV-specific CD8+ T cells in HIV controllers. Cells were gated (A) on live cells using live/dead stain cells kit, (B) on lymphocytes, (C) on CD3+/CD8+ expressing cells, (D) on HIV-specific CD8+ T cells using tetramer staining, (E) on different subset of CD38 and HLA-DR expressing cells using isotypes as control. (TIF) [file pone.0101920.s001.tif]

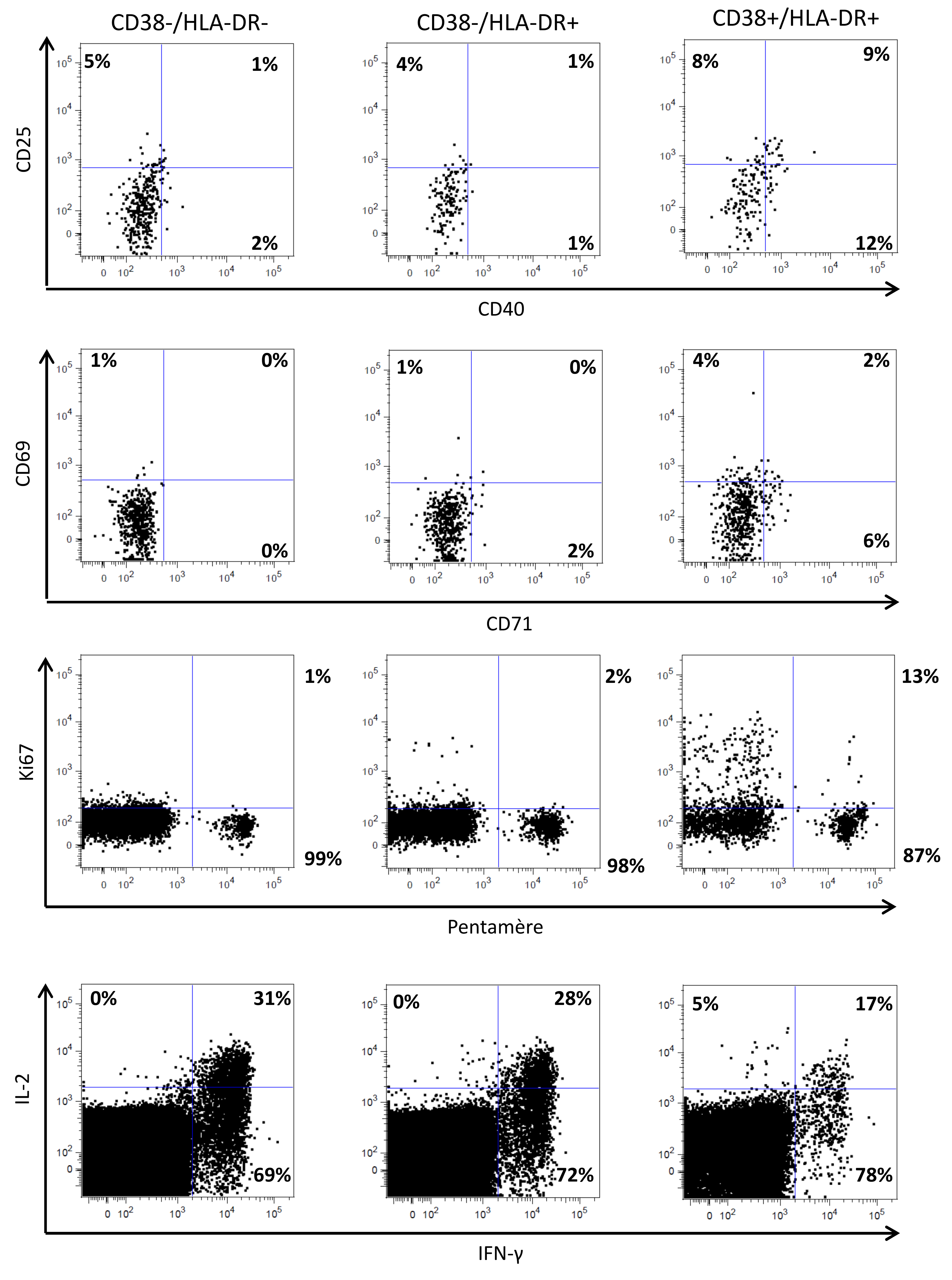

Supplement: Figure S2 — Dot plots of activation marker expression and cytokine production on different subsets of HIV-specific CD8+ T cells in HIV controllers. Representative dot plots of CD25 and CD40 (A), CD69 and CD71 (B), Ki67 (C) expression and IL-2 and/or IFN-γ secretion (D) on CD38−/HLA-DR− (left panel), CD38−/HLA-DR+ (middle panel) and CD38+/HLA-DR+ (right panel) subsets (TIF) [file pone.0101920.s002.tif]

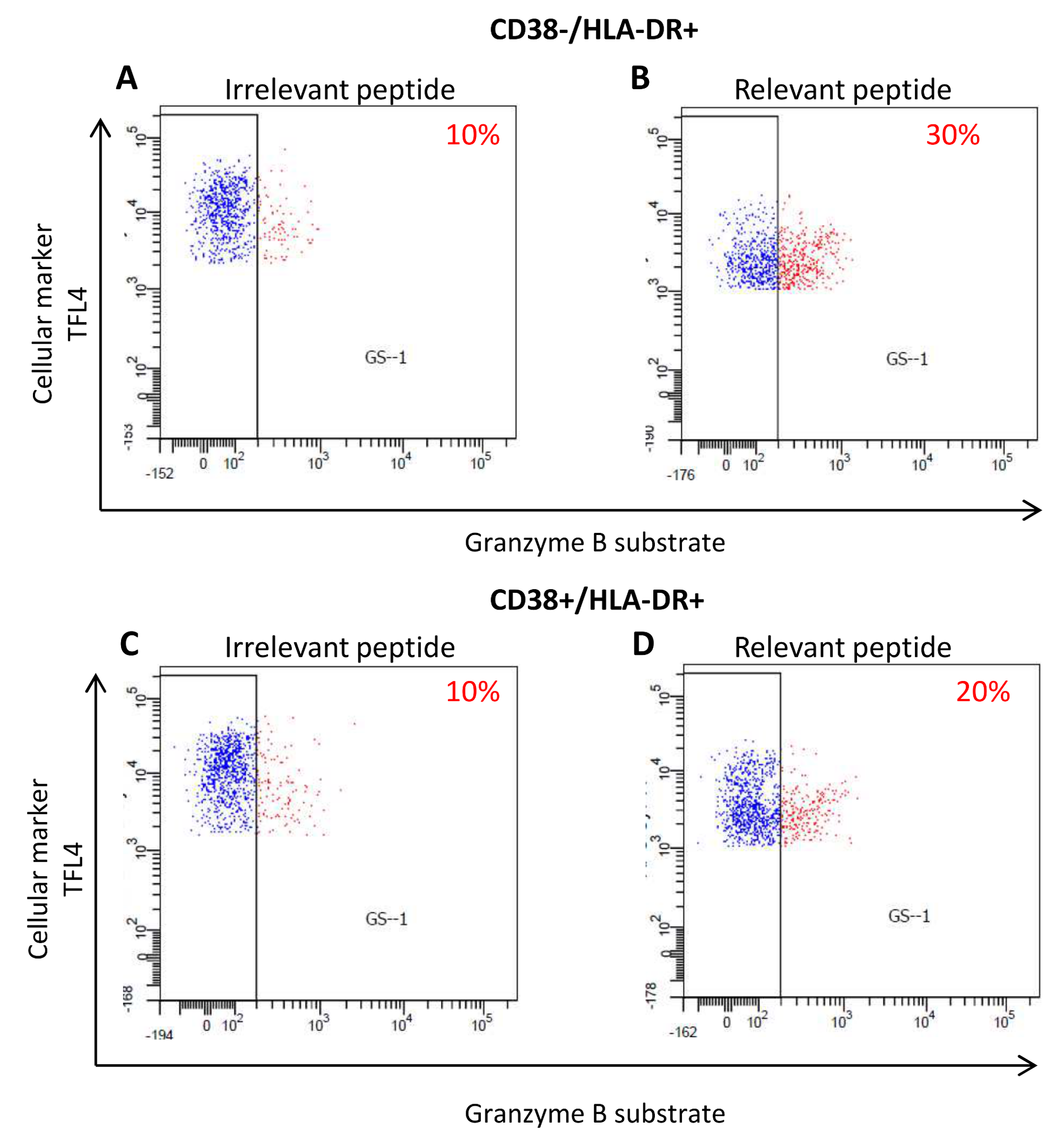

Supplement: Figure S3 — Dot plots of the cytotoxic assay of CD38−/HLA-DR+ and CD38+/HLA-DR+ HIV-specific CD8+ T cells. Cytotoxic capacity was assessed by measuring the frequency of positive target cells for granzyme B substrate. The results were expressed as the difference between the frequency using effector cells co-cultured with target cells incubated with the relevant peptide (B and D) and the frequency in the negative control using effector cells co-cultured with target cells incubated with irrelevant peptide (A and C). (TIF) [file pone.0101920.s003.tif]
